# *FOXP3 Transcription Factor*: A Candidate Marker for Susceptibility and Prognosis in Triple Negative Breast Cancer

**DOI:** 10.1155/2014/341654

**Published:** 2014-04-30

**Authors:** Leandra Fiori Lopes, Roberta Losi Guembarovski, Alda Losi Guembarovski, Marina Okuyama Kishima, Clodoaldo Zago Campos, Julie Massayo Maeda Oda, Carolina Batista Ariza, Karen Brajão de Oliveira, Sueli Donizete Borelli, Maria Angelica Ehara Watanabe

**Affiliations:** ^1^Laboratory of Molecular Genetics and Immunology, Department of Pathological Sciences, Biological Science Center, State University of Londrina, 86057-970 Londrina, PR, Brazil; ^2^Department of Pathology, Clinical Analysis and Toxicology, Health Sciences Center, State University of Londrina, 86057-970 Londrina, PR, Brazil; ^3^Department of Medical Clinic, Health Sciences Center, State University of Londrina, 86057-970 Londrina, PR, Brazil; ^4^Department of Basic Health Sciences, Maringá State University, 87020-900 Maringá, PR, Brazil

## Abstract

Triple negative breast cancer (TNBC) is a relevant subgroup of neoplasia which presents negative phenotype of estrogen and progesterone receptors and has no overexpression of the human epidermal growth factor 2 (HER2). FOXP3 (forkhead transcription factor 3) is a marker of regulatory T cells (Tregs), whose expression may be increased in tumor cells. This study aimed to investigate a polymorphism (rs3761548) and the protein expression of FOXP3 for a possible involvement in TNBC susceptibility and prognosis. Genetic polymorphism was evaluated in 50 patients and in 115 controls by allele-specific PCR (polymerase chain reaction). Protein expression was evaluated in 38 patients by immunohistochemistry. It was observed a positive association for homozygous AA (OR = 3.78; 95% CI = 1.02–14.06) in relation to TNBC susceptibility. Most of the patients (83%) showed a strong staining for FOXP3 protein in the tumor cells. In relation to FOXP3-positive infiltrate, 47% and 58% of patients had a moderate or intense intratumoral and peritumoral mononuclear infiltrate cells, respectively. Tumor size was positively correlated to intratumoral FOXP3-positive infiltrate (*P* = 0.026). In conclusion, since FOXP3 was positively associated with TNBC susceptibility and prognosis, it seems to be a promising candidate for further investigation in larger TNBC samples.

## 1. Introduction

The National Cancer Institute (INCA) estimated 52,680 new cases of breast cancer for 2012 and 2013 in Brazil. It is worth noting that, regardless of nonmelanoma skin cancer, the mammary tumor is the most common among women in many regions of Brazil, accounting high morbidity and mortality among Brazilian women [1].

Breast cancer represents a complex and heterogeneous disease that comprises distinct pathologies, histological features, and clinical outcome. The status of estrogen receptor (ER), progesterone receptor (PR), and human epidermal growth factor receptor type 2 (HER2) has been used as predictive markers to identify a high-risk phenotype and for selection of the most efficient therapies [2, 3]

Triple-negative breast cancer (TNBC) is a subtype characterized by the lack of ER, PR, and HER2 expression and is associated with younger age at diagnosis and often occurs in African-American, premenopausal, and overweighed women (particularly with abdominal obesity) [4]. It represents approximately 12–17% of all breast cancers [5] and encompasses a heterogeneous group of tumors including, but not limited to, those classified as basal-like. TBNC is aggressive, showing a tendency towards early metastasis and having a poor overall outcome despite being highly responsive to conventional chemotherapy. The aggressive clinical course, poor prognosis, and lack of specific therapeutic options for this subtype of tumor have intensified current interest in this group of patients [6].

Regulatory T cells (Treg) represent a heterogeneous population of cells composed of discrete subsets with different phenotypes and functions [7]. The most specific marker to identify Tregs is* FOXP3*, a member of the forkhead-winged helix family of transcription factors [8, 9] that plays a role in various cellular processes.* FOXP3* expression in tumors was associated with worse overall survival and this gene was also considered a strong prognostic factor for distant metastases-free survival but not for local recurrence risk [10]. Hirata et al. reviewed some well-established molecular markers of therapeutic value in breast cancer and also promising new markers not routinely used in clinical practice, which includes* FOXP3* gene [11].

Promoter regions are potential candidates for the presence of functional single nucleotide polymorphism (SNPs), as they are involved in transcription initiation, and many of the cis-acting elements that regulate gene expression possibly harbor functional polymorphisms [12]. As recently reviewed by Oda et al., FOXP3 polymorphisms occur with high frequency in the general population and have been studied in common multifactorial human diseases, like diabetes, allergic rhinitis, and breast cancer [13]. It is known that SNPs in the promoter region of* FOXP3* gene may affect its expression [14]. Since it has been previously shown that* FOXP3* is involved in breast cancer development [15], several studies have been conducted to investigate a SNP (*rs3761548, C/A)* in the promoter region of* FOXP3* in patients with this neoplasia [16, 17], but its exact role is not yet well understood.

In this context, the present report attempts to investigate if there is an association between genetic polymorphism and protein expression of* FOXP3* gene with clinical outcome, in a search for its involvement in pathogenesis of TNBC.

## 2. Material and Methods

### 2.1. Human Subjects

Retrospectively (10 years), clinic and pathological information (tumor size, lymph node involvement, and nuclear grade) and tissue samples of 50 TNBC were obtained at Cancer Hospital of Londrina (HCL), Londrina, Paraná State, Brazil. Clinical staging was determined according to the Union of International Control of Cancer (UICC) classification criteria.

For control group, blood samples from 115 women neoplasia-free were collected in the Blood Center of North Parana, Brazil. The protocol was approved by the Institutional Human Research Ethics Committee of the State University of Londrina, Paraná, Brazil (CAAE No. 0179.0.268-09-CONEP 268).

### 2.2. DNA Extraction

For patients, the genomic DNA was isolated from formalin-fixed paraffin embedded samples according to Isola et al. [18] protocol. For neoplasia-free control group, the DNA was obtained from peripheral white blood cells using Biopur kit (Biometrix, Curitiba, PR, Brazil). The DNA was resuspended in 50 *μ*L of Milli-Q water and quantified by NanoDrop 2000c Spectrophotometer (NanoDrop Technologies, Wilmington, DE, EUA) at a wavelength of 260/280 nm.

### 2.3. Genetic Polymorphism of* FOXP3 rs3761548*

DNA (100 ng) was amplified by polymerase chain reaction (PCR) with specific primers for* FOXP3 *following the GenBank accession number NT_079573.4. The samples were amplified using the buffer kit plus 1.25 units Taq polymerase (Invitrogen, Carlsbad, CA, USA).

PCR conditions were 10 min denaturation at 94°C, 35 cycles of 45 s at 94°C, 1 min at 67°C and 1 min at 72°C, and 10 min elongation at 72°C in a thermocycler (PCR-Sprint Hybaid-Guelph, Ontario, Canada). Amplicons of 334 base pairs for A allele and 333 base pairs for C allele were analyzed by electrophoresis on acrylamide gel (10%) and detected by a nonradioisotopic technique using a commercially available silver staining method (Table 1).

### 2.4. Immunohistochemical Staining

For immunohistochemical analysis, 5 *μ*m of tissue sections was obtained from breast tumors samples. Samples were heated at 56°C, deparaffinized in xylene, and rehydrated in a graded alcohol series. Antigen retrieval was performed with citrate buffer and a mouse/rabbit monoclonal antibody for human* FOXP3* (clone 236A/E7; Abcam, Cambridge, UK; eBioscience) was used. The sections were stabilized at room temperature for 30 min and washed with PBS (phosphate buffered saline) and anti-mouse/rabbit HRP secondary antibody was used as second step (Bio SB Inc. Santa Barbara, CA, USA). The diaminobenzidine (DAB) chromogen system was used (Sigma-Aldrich, USA) and counter staining was performed with Gill's hematoxylin and slide mounts in Canada balsam. Controls were performed to verify the specificity of primary antibody and all analyses were made with at least two pathologists.

### 2.5. Statistical Analysis

The case control association study was performed using contingency tables to calculate the odds ratios (OR) with a confidence interval (CI) of 95 %. A 3x2 contingency table was constructed, considering wild type genotype (OR = 1.0) as reference, to determine the OR value for heterozygotes and rare genotypes. GraphPad Prism version 5.00 for Windows was used (GraphPad Software, San Diego, CA, USA). The rare homozygous and heterozygotes for* FOXP3* gene were grouped for the presence of at least one allelic variant, in a dominant model of analysis.

Spearman correlation and Chi square statistical tests were used to analyze immunohistochemistry and genetic polymorphism in relation to clinical outcome, using SPSS Statistics 17.0 software (SPSS inc., Chicago, IL, USA). A *P* value** <**0.05 was considered statistically significant.

## 3. Results

The mean age of patients was 54 ± 13 years. For some patients specific clinic pathological characteristics were not available. It was observed from patients who had the respective information; 83% presented nuclear grade in stage II or III, 51% had lymph node commitment, and the tumor size mean was 3.5 cm.

### 3.1. Genetic Polymorphism Analysis

The rs3761548 polymorphism of* FOXP3* gene was evaluated in 50 TNBC patients and in 115 neoplasia free controls. The genotype frequency was 12% (6/50) and 3.48% (4/115) for AA homozygote, 34% (17/50) and 57.39% (66/115) for CA heterozygote, and 54% (27/50) and 39.13% (45/115) for CC homozygote, in patients and controls, respectively (Table 2). Case control study indicated a positive association for AA homozygous genotype in relation to TNBC susceptibility (OR = 3.78, 95% CI = 1.02 to 14.06).

When comparing genotypes of* FOXP3* and clinical outcome, there was no significant association with tumor size (*P* = 0.482; rho = 0.102), lymph node involvement (*P* = 0.890; rho = −0.023), and nuclear grade (*P* = 0.682; rho = −0.062).

### 3.2. Immunohistochemistry Analysis

**Figure 1:**
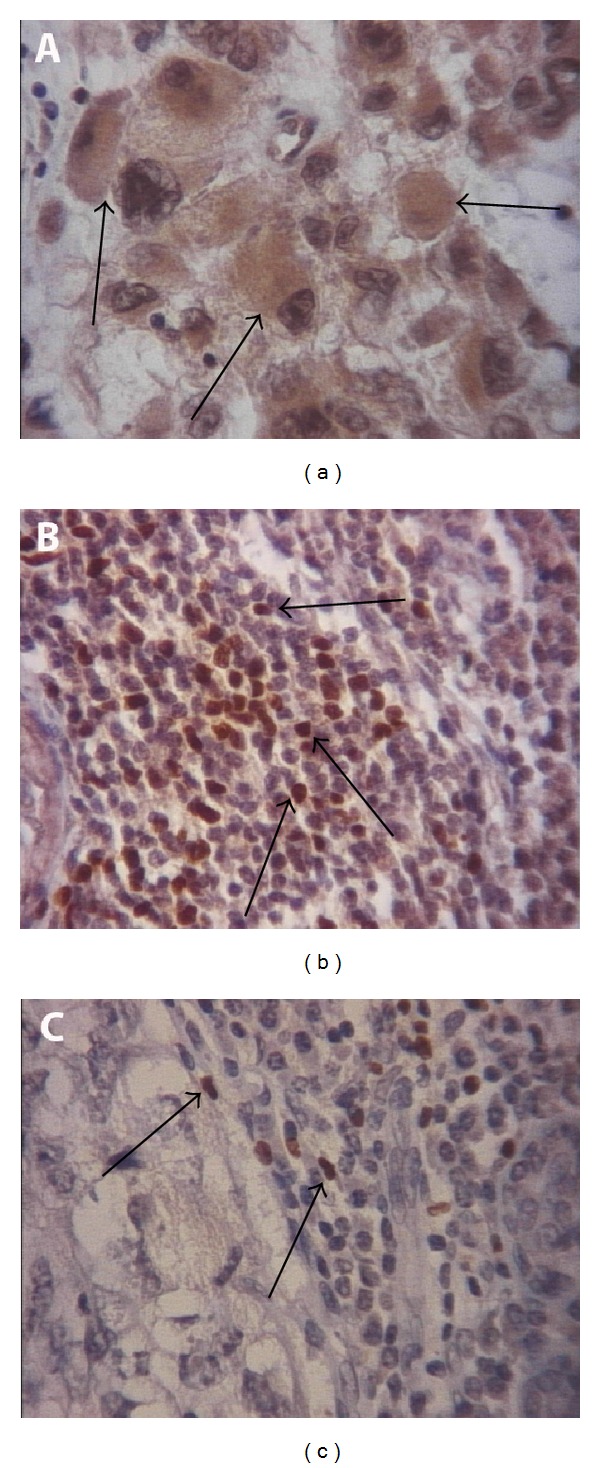
FOXP3 expression by immunohistochemistry in TNBC tissue samples. (a) FOXP3 cytoplasmic expression in breast tumor cells; (b) FOXP3 intratumoral mononuclear infiltrating cells in breast tumor; and (c) FOXP3 peritumoral mononuclear infiltrating cells in breast tumor. The arrows indicated some strong staining. Magnification 400x.

In 38 patients analyzed for FOXP3 protein expression, “cytoplasmic” tumoral staining was verified predominantly in all tissue samples analyzed (Figure 1(a)). Most of TNBC patients (83%) had high expression of tumoral FOXP3 protein (two or three crosses). Additionally, for patients who were lymph node free of neoplasia, a strong FOXP3 expression was verified, most of them being with a strength signal (three crosses), despite being not statistically significant (*P* = 0.14).

Tumors sizes and nuclear degrees are equally distributed among the patients according to FOXP3 protein expression (*P* = 0.42 and *P* = 0.12), respectively. Allelic variant showed no correlation with FOXP3 protein expression (*P* = 0.792, rho = −0.046). Therefore, despite being not significant, it was observed that allele A carriers for* FOXP3* gene present higher tumoral expression of this protein by immunohistochemistry (*P* = 0.078).

Table 3 described the protein expression analysis in relation to infiltration of mononuclear cells positive for FOXP3 staining in tumor microenvironment of 38 patients. 47% and 58% of the sample presented a moderate or intense intratumoral and peritumoral infiltrated, respectively (Figures 1(b) and 1(c)). There were no significant results for both mononuclear infiltrated (intratumoral and peritumoral) in relation to lymph node involvement or nuclear grade parameters. Therefore, the results indicated a significant association between intratumoral infiltrated and tumor size (*P* = 0.026). It was observed that this significance was attributed to tumors between 1.5 and 3 cm, since this prognostic parameter was divided into three categories based on clinical criteria (less than 1.5 cm, 1.5 to 3 cm, and more than 3 cm).

## 4. Discussion

Breast cancer is a complex disease with high clinical morphological and biological heterogeneity. It is known that mammary tumors with similar clinical histology and different prognoses had different therapeutic responses [19–21].

The* FOXP3* gene expressed in CD4+ CD25+ Tregs in normal physiological conditions encodes the FOXP3 protein, which regulates the activation of T cell, works as a transcriptional repressor and downregulates cytokines expression in T cells [8, 22].

The autoimmune disease that lacks functional* FOXP3*, observed in human and in mice, indicates that this transcription factor has a crucial role in the regulation of T-cell function [23]. Additionally, it has been suggested that* FOXP3*-positive cells in tumors could be a novel therapeutic target that could improve outcomes for such patients [24]. So the high rate of somatic mutations in breast tumors, its conserved sequence, and the regulation of important pathways make* FOXP3* a very plausible candidate for a susceptibility gene in cancer [16].

In this study, we analyzed a* FOXP3* polymorphism (rs3761548) in 50 TNBC patients and in 115 controls free of neoplasia (Table 2). The results indicated a positive association for AA homozygous genotype in relation to TNBC development (OR = 3.78, 95% CI = 1.02 to 14.06). Therefore, we suggested that individuals who had inherited both copies of this allelic variation had a higher susceptibility for developing this subtype of breast cancer than individuals with other genotypes. As far as we researched, there is no articles relating genetic polymorphism of* FOXP3* and TNBC susceptibility in a Brazilian population, but positive associations have been proposed with other diseases such as psoriasis [25] and allergic rhinitis [26]. Raskin et al. [16] investigated three genetic polymorphisms in the* FOXP3* gene in patients with breast cancer, but not triple-negative subtype, and found none significant associations. Additionally, these authors postulated that* FOXP3* gene may be involved with the hereditary breast cancer form, with high penetrance mutations. Our results are not in accordance with these authors, since we found a positive association between a specific* FOXP3* polymorphic mutation and TNBC susceptibility (Table 2). Despite the low number of homozygotes observed in both groups, we found 12% of AA homozygotes in TNBC group versus only 4% in the control group, although the last one is composed of a much larger number of individuals. Thus, although our results deserve caution by the sample size, they indicate a possible role for* FOXP3* gene in TNBC susceptibility.

Initially, it was postulated that* FOXP3* expression was thought to be restricted to hematopoietic tissues. However, although data are scant,* FOXP3* expression in other tissues has also been observed, including human tumor cells [27]. Therefore, biological functions of* FOXP3* in tumor cells and its significance presently remain unclear. According to the same authors, their study clearly demonstrates that FOXP3 expression is not restricted to pancreatic carcinoma cells but seems to characterize many other tumors not only of epithelial (e.g., lung, breast, and colon) but also of other tissue origins (melanoma, leukemia).

Recent data suggest that* FOXP3* expression in tumor cells could be an independent strong prognostic factor for distant metastases in breast cancer [28], but in contrast with these data,* FOXP3* was also recently demonstrated to be a tumor suppressor gene, acting as a transcriptional repressor of* SKP2* and* HER2*, two breast cancer important oncogenes [15, 29]. In the present work, we analyzed the tumoral protein expression of FOXP3 and found that 83% of the patients had a strong expression of this protein in the tumor microenvironment. Other studies found that cancer cells were FOXP3 positive in 57% of HER2+ breast tumors [30] and in 66% of archival samples from human breast cancer patients [10], indicating that our sample of TNBC demonstrated a high expression of this protein and that FOXP3 may have different expression levels in subtypes of breast cancer, with specific prognostic implications.

According to Triulzi et al., in contrast to a putative oncosuppressor role for* FOXP3*, emerging evidence from studies of human cancer samples points to its prometastatic action in vivo, based on the correlation between its expression by tumor cells and poor prognosis. The authors point out that, overall, the associations between FOXP3 expressions in tumor cells are with poor patient's prognosis [31]. Kim et al. found FOXP3 expression in 27.9% of their breast cancer samples and the positive tumors were associated with significantly higher nuclear grade, higher histologic grade, and a more negative estrogen receptor status. A multivariate analysis with adjustment for patient age and human epidermal growth factor receptor 2 status demonstrated significantly poor survival of FOXP3-strong-positive patients in node-positive patients, which suggest that this protein expression in breast cancer cells is associated with poor prognosis [32].

In our sample we did not observe any associations between protein expression of FOXP3 and clinical outcome parameters, considering tumor size, lymph node involvement, and nuclear grade. Ladoire et al. also found no association with tumor size and lymph node involvement; however, the authors observed a significant result between protein expression and tumor grade (*P* = 0.046), which strengthens prognostic differences for FOXP3 protein expression in mammary tumor subgroups [30].

Another relevant point is that FOXP3 protein expression observed in our TNBC sample was predominantly cytoplasmic. According to the literature data, a cytoplasmic localization was observed in human cancer cells in various tissues [27, 33], including breast carcinoma [10, 25] and breast adenocarcinoma cell line (MCF7) [27]. Also, in a study concerning* FOXP3* expression in prostate cancer cells [34], the authors demonstrated that genetic mutations in this gene could be detected in cancer cells and restrained its expression in the cytoplasm. According to Triulzi et al., in most breast carcinomas, FOXP3 staining was localized predominantly in the cytoplasm, although both cytoplasmic and nuclear expressions were present in some specimens and a few showed only nuclear staining [31]. In this TNBC sample, we also found that most patients had cytoplasmic expression of FOXP3 protein, but some had concomitant perinuclear and/or nuclear expression.

It is noteworthy that experimental evidences show that* FOXP3* downregulates the oncogene* HER2* and other genes in this signaling pathway. Within this context, our results concerning FOXP3 protein expression are in accordance, since we observed a high expression in tumor cells of TNBC patients, who are exactly negative for* HER2* expression by immunohistochemistry. Therefore, Karanikas et al. that found a high expression of FOXP3 in MCF7 and other cell lines said that whether this expression by tumor cells is directly related to carcinogenesis or results indirectly by activation of its normally silent gene is questionable [27].

Although T cells present the most important immunological response in tumor growth in early stages of cancer, they become Tregs after chronic stimulation and interactions with tumor cells, promoting rather than inhibiting cancer development and progression [35]. Karanikas et al. point that the highest mRNA expression levels of FOXP3 observed by tumor cells were with the breast cancer line MCF7, which expressed at least half as much FOXP3 as a Treg clone did and at least ten times more than a population of PHA blasts. This expression level indicates that FOXP3 transcripts are present in a sufficiently high number in tumor cells and caution should be exerted when detection of* FOXP3* mRNA expression in surgical tumor samples is used as an index of tumor infiltration by Tregs [27].

The study of Demir et al. established a predictive and prognostic effect of intratumoral FOXP3 Tregs in locally advanced breast cancer patients. The authors point out that to predict clinical outcome, an evaluation of FOXP3+ Tregs in tumoral tissues before and after neoadjuvant chemotherapy should be considered for these high-risk patients [36]. Concerning infiltrate of mononuclear cells expressing* FOXP3 *in the tumor microenvironment, we had the results of 38 patients and observed that 47% and 58% of these had a moderate or intense intratumoral and peritumoral infiltrated, respectively.

Gokmen-Polar et al. found that the number of FOXP3-expressing T regulatory cells does not differ significantly between sentinel nodes with and without metastatic breast carcinoma and also does not affect primary tumor characteristics like tumor type, grade, size, hormone receptor, and HER2 status [37]. Ladoire et al. in their series of HER2+ over expressing breast carcinoma found that the presence of* FOXP3* Treg infiltration had no prognostic behavior [30]. Corroborating these data, we also did not find any significant results when analyzing this parameter in relation to lymph node involvement and nuclear grade.

On the other hand, we observed a significant association with tumor size parameter (*P* = 0.026) and that this significance was attributed to tumor size range from 1.5 to 3 cm. On a multivariate analysis Lee et al. showed that FOXP3-positive Tregs were an independent prognostic factor for overall survival and progression-free survival with hazard ratios of 2.4 (95% CI 1.0–5.6; *P* = 0.049) and 2.0 (95% CI 1.1–3.6; *P* = 0.032), respectively. So these authors concluded that in TNBC patients FOXP3-positive Tregs had stronger prognostic significance. The finding of improved survival associated with highly infiltrating FOXP3-positive Tregs in TNBC contrasted with several other types of solid cancers, but according to them, TNBC may be differently driven by FOXP3 via an immune mechanism [35]. In this context, we hypothesized that Tregs* FOXP3 *positive, which were present in the tumor, could act as stimulator of growing in intermediates tumors size (1.5 to 3 cm), in relation to small tumors (less than 1.5 cm). Likewise, another mechanism could stimulate even larger sizes of tumors TNBC (above 3 cm), since, in our sample, the intratumoral infiltrate does not appear to be positively associated with larger tumors.

## 5. Conclusion

Since we found a significant association between a specific genetic variant in* FOXP3* gene and a high expression of this protein in the tumor microenvironment, which would agree with the fact that TNBC patients do not present the overexpression of* HER2 *oncogene, and also a positive correlation between* FOXP3*-positive infiltrate and the prognostic parameter tumor size, we suggest that this transcript factor could be a promising marker of susceptibility and prognosis in human breast cancer pathogenesis, especially in the triple-negative molecular subtype.

## Figures and Tables

**Table 1:** Oligonucleotides and amplicons for *FOXP3* gene.

Gene	Allele	Primer sequence	PCR product
*FOXP3* (C/A, rs3761548)	A	5′-CTG GCT CTC TCC CCA ACT GA-3′	334 bp
		5′-ACA GAG CCC ATC ATC AGA CTC TCT A-3′	
	C	5′-TGG CTC TCT CCC CAA CTG C-3′	333 bp
		5′-ACA GAG CCC ATC ATC AGA CTC TCT A-3′	

**Table 2:** Genotype distribution and case control study for *FOXP3* gene in patients and controls.

		Controls (*n* = 115)	Patients (*n* = 50)	OR	IC	*P* value (*χ* ^2^)
*FOXP3* rs3761548	CC	45 (39%)	27 (54%)	1.00	—	—
	CA	66 (57%)	17 (34%)	0.38*	0.19–0.76	0.006*
	AA	4 (4%)	6 (12%)	3.78*	1.02–14.06	0.035*
	CA + AA	70 (61%)	23 (46%)	0.55	0.28–1.07	0.077

*P < 0.05.

**Table 3:** FOXP3 protein expression in mononuclear cells in relation to prognostic parameters of TN breast tissues.

FOXP3 protein expression (*n* = 38)	Intensity and prognostic parameters	Frequency (%) or *P* value
Intratumoral infiltrated of mononuclear cells	Moderate/intense	47%
	Lymph node involvement	0.310
	Nuclear grade	0.531
	Tumor size	0.026*

Peritumoral infiltrated of mononuclear cells	Moderate/intense	58%
	Lymph node involvement	0.679
	Nuclear grade	0.309
	Tumor size	0.598

*P < 0.05.
